# Meta-analysis of prophylactic corticosteroid use in post-ERCP pancreatitis

**DOI:** 10.1186/1471-230X-8-6

**Published:** 2008-02-14

**Authors:** Minghua Zheng, Jianling Bai, Bosi Yuan, Feng Lin, Jie You, Mingqin Lu, Yuewen Gong, Yongping Chen

**Affiliations:** 1 Department of Infection and Liver Diseases, The First Affiliated Hospital of Wenzhou Medical College, Wenzhou, China; 2 Department of Epidemiology and Biostatistics, School of Public Health Nanjing Medical University, Nanjing, China; 3 Department of Gastroenterology, Clinical School of Nanjing, Southern Medical University, Jinling Hospital, Nanjing, China; 4 Department of Gynecology, The First Affiliated Hospital of Wenzhou Medical College, Wenzhou, China; 5 Department of Oncology, The First Affiliated Hospital of Wenzhou Medical College, Wenzhou, China; 6 Faculty of Pharmacy, University of Manitoba, Winnipeg, Canada

## Abstract

**Background:**

Acute pancreatitis is a common complication of endoscopic retrograde cholangiopancreatography and benefit of pharmacological treatment is unclear. Although prophylactic use of corticosteroid for reduction of pancreatic injury after ERCP has been evaluated, discrepancy about beneficial effect of corticosteroid on pancreatic injury still exists. The aim of current study is to evaluate effectiveness and safety of corticosteroid in prophylaxis of post-endoscopic retrograde cholangiopancreatography pancreatitis (PEP).

**Methods:**

We employed the method recommended by the Cochrane Collaboration to perform a meta-analysis of seven randomized controlled trials (RCTs) of corticosteroid in prevention of post-ERCP pancreatitis (PEP) around the world.

**Results:**

Most of the seven RCTs were of high quality. When the RCTs were analyzed, odds ratios (OR) for corticosteroid were 1.13 [95% CI (0.89~1.44), p = 0.32] for PEP, 1.61 [95% CI (0.74~3.52), p = 0.23] for severe PEP, 0.92 [95% CI (0.57~1.48), p = 0.73] for post-ERCP hyperamylasemia respectively. The results indicated that there were no beneficial effects of corticosteroid on acute pancreatitis and hyperamylasemia. No evidence of publication bias was found.

**Conclusion:**

Corticosteroids cannot prevent pancreatic injury after ERCP. Therefore, their use in the prophylaxis of PEP is not recommended.

## Background

ERCP is one of the important procedures for diagnosis and treatment of several biliary and pancreatic conditions. However, ERCP can also cause acute pancreatitis and result in significant morbidity and mortality [1,2]. Depending on the definition, it has been reported that incidence of post-ERCP pancreatitis (PEP) was 1% to 40% of cases, whereas post-operative hyperamylasemia can be up to 70% of cases [3]. Although most cases of PEP were mild, about 10% of them were severe pancreatitis [4], which could result in prolonged stay in hospitals and increase risk to patients' life.

There were numerous attempts to minimize occurrence and severity of PEP. However most of them were disappointed. Prevention of PEP with calcitonin, aprotonin, nifedipine and glucagon did not show any decrease in post-ERCP hyperamylasemia or pancreatitis [5-8]. Moreover, a few studies with long-acting somatostatin analogue – octreotide produced conflicting results [9-12]. Furthermore, a recent meta-analysis of all prospective randomized clinical trials (RCTs) of protease inhibitor – gabexate mesilate concluded that gabexate mesilate cannot prevent the pancreatic injury after ERCP [13].

Corticosteroid is an anti-inflammatory hormone and is able to ameliorate injury response. It has been shown that corticosteroid is able to increase activity of C1-antiprotease inhibitor [14], decrease synthesis of phospholipase A2 [15], and suppress cellular and humoral responses. Since acute pancreatitis is a process of autodigestion characterized by release of activated pancreatic enzymes [16], corticosteroid may reduce inflammatory response during initial steps of autodigestive cascade. Therefore, one large retrospective and 7 prospective studies of corticosteroid in prophylaxis of PEP have been conducted recently [17-24]. However, these studies could not reach a clear conclusion about beneficial effects of corticosteroid on prophylaxis of PEP. Therefore, it will be necessary to conduct a meta-analysis of all available RCTs to reveal use of corticosteroid in prophylaxis of PEP.

## Methods

### Selection criteria

We searched different databases, which included the Cochrane Controlled Trials Register on The Cochrane Library Issue 2, 2007, MEDLINE (January, 1966 – June, 2007), EMBASE.com (January, 1966 – June, 2007) and the China Biological Medicine Datadase (CBMdisc) (January, 1978 – June, 2007) by the terms of *pancreatitis*, *ERCP*, prevent*, *corticosteroid*, *prednisone*, *PEP*. The reference lists of pertinent reviews and retrieved articles had been checked for additional study identification.

In the meta-analysis, the following inclusive selection criteria were set and reviewed by two independent investigators: (1) each trial should be a prospective randomized controlled clinical trial, (2) the age of patient population should be over 18 years, (3) the patients were scheduled to undergo ERCP and/or endoscopic sphincterotomy, (4) randomized comparisons of corticosteroid versus placebo should be included regardless of initial time of treatment, treatment duration, dose and administration route of drug, (5) co-interventions (including treatment of complications) were allowed if administered equally to all intervention groups. The following exclusive selection criteria were set: (1) quasi-randomized trials and non-randomized studies, (2) active acute pancreatitis, (3) difference of co-interventions between intervention arms, (4) repetitive reports (if more than one version of the same study was retrieved, only the most recent one was used).

**Figure 1:**
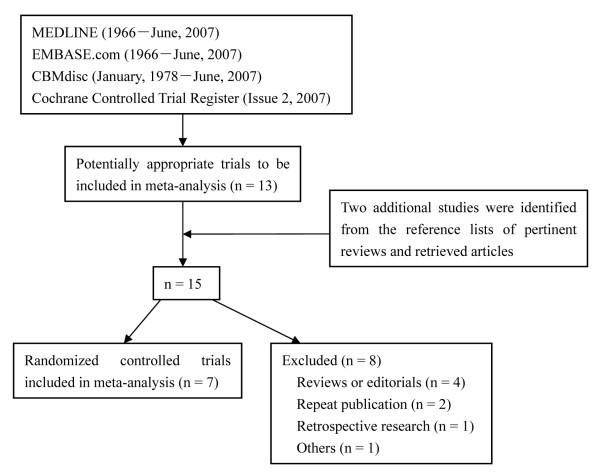
Identification of eligible randomized controlled trials from different medicine databases.

A total of 15 clinical trials and reports has been identified and only seven trials [18-24] were qualified by our selection criteria (Figure 1). Four out of seven studies included patients submitted only to diagnostic ERCP and three to diagnostic and therapeutic ERCP. All of the studies adopted the PEP diagnostic criteria that Cotton et al. [3] proposed in 1991 and a severe PEP was reported in five studies. Five out of seven RCTs were adequate in the allocation concealment judgment and two were unclear. The studies were independently evaluated by two of us with five outcomes, which included three primary outcomes (PEP, severe PEP and case-fatality ratio of PEP) and two secondary outcomes (post-ERCP hyperamylasemia and abdominal pain). Discrepancies in the evaluation of some studies were resolved through discussion between the reviewers. Main features of the trials included in meta-analysis are shown in Table 1.

**Table 1:** Randomized controlled trials on the use of corticosteroid for the prevention of pancreatic injury after ERCP

Author and publication year	Setting	Jadad score	Sample size	Type of corticosteroid administration	Dosage (mg)	Duration
Budzynska, 1997	Poland single centre	1	184	Prednisone, orally	40	15 hours and 3 hours before ERCP
Dumot, 1998	United States multicentre	4	255	Methylprednisolone, intravenous bolus	125	15 to 30 minutes before ERCP
De Palma, 1999	Italy single centre	5	529	Hydrocortisone, intravenous infusion	100	Immediately before ERCP
Budzynska, 2001	Poland single centre	2	201	Prednisone, orally	40	15 hours and 3 hours before ERCP
Manolakopoulos, 2002	Greece multicentre	5	228	Hydrocortisone, intravenous infusion	100	30 minutes before ERCP
Sherman, 2003	United States multicentre	4	1115	Prednisone, orally	40	15 hours and 3 hours before ERCP
Kwanngern, 2005	Thailand single centre	4	120	Hydrocortisone, intravenous infusion	100	1 hour before ERCP

### Assessment of study quality

Quality of included reports was scored using the Jadad composite scale [25], which assesses descriptions of randomization, blinding, and dropouts (withdrawals) in reports [26]. The quality scale ranges from 0 to 5 points with a low-quality report of score at 2 or less and a high-quality report of score at least 3 [27].

### Statistical analysis

The meta-analysis was carried out by a biostatistician (Jianling Bai) according to the Cochrane Reviewers' Handbook recommended by The Cochrane Collaboration. Pooled odds ratio (OR) was calculated using the general inverse variance (IV) fixed-effect model. The heterogeneity between studies was examined by DL Q statistic [28]. If results were heterogeneous (p < 0.05), a random-effects model was employed using the DerSimonian and Laird (DL) methods. For studies in which the constructed 2×2 tables contained cells with zero events, a standard correction factor of 0.5 was added to each cell. Pooled OR was presented as standard plots with 95 percent confidence intervals (CI). Publication bias was measured by Begg and Mazumdar's proposed adjusted rank correlation test [29] and Egger's linear regression approach [30], and was shown as a funnel plot. Sensitivity-analysis was also performed to assess the reliability of meta-analysis. The statistical package RevMan version 4.2 (provided by The Cochrane Collaboration, Oxford, England) was used for statistical analysis.

## Results

### Primary outcome

**Figure 2:**
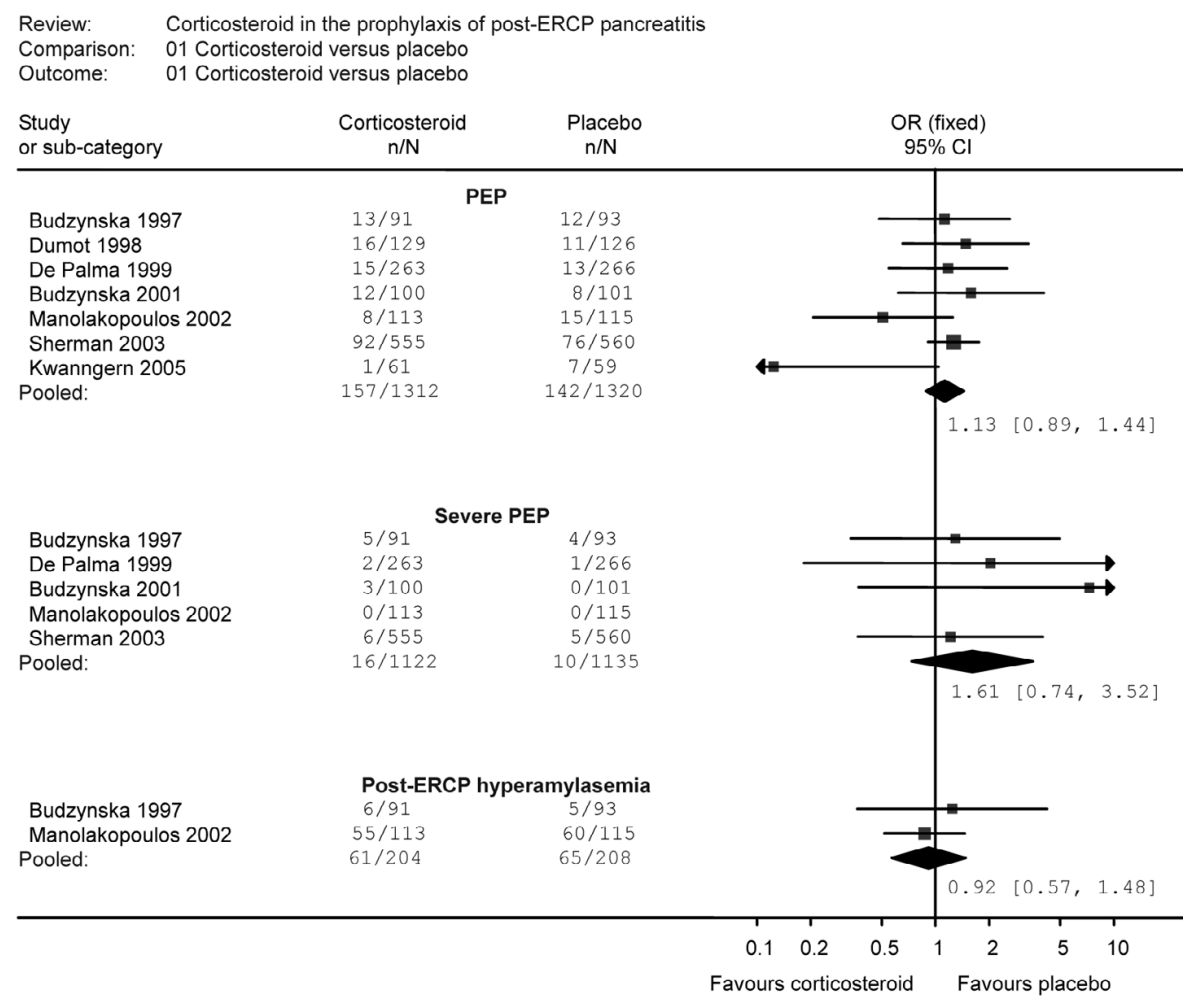
Effect of corticosteroids in the prophylaxis of post-ERCP pancreatitis.

In this report, we considered PEP as the primary outcome which was divided into general PEP and severe PEP. The report of general PEP was noticed in all seven RCTs [18-24]. These trials included 2632 patients with 299 patients suffering from PEP. Among PEP-suffering patients, 157 (12.0%) patients were treated with corticosteroid whereas 142 (10.8%) patients were treated with placebo. There was no significant heterogeneity among these studies (χ^2 ^= 8.56, 6 degrees of freedom, p = 0.20). However, analysis by a fixed-effects model indicated an IV fixed-effect pooled OR = 1.13 [(95 percent CI 0.89 to 1.44); p = 0.32] with no significant association between the use of corticosteroid and the reduction of PEP (Figure 2, Table 2). When stratified by setting, there was no significant reduction of PEP in either single centre studies (IV fixed-effect pooled OR = 1.04 [(95 percent CI 0.66 to 1.64); p = 0.87]) or multicentre studies (IV fixed-effect pooled OR = 1.17 [(95 percent CI 0.88 to 1.56); p = 0.29]) (Table 3). There was also no significant heterogeneity in either single centre studies (χ^2 ^= 4.75, 3 degrees of freedom, p = 0.19) or multicentre studies (χ^2 ^= 3.84, 2 degrees of freedom, p = 0.15). Moreover, when studies were stratified by Jadad score, there was no significant reduction in PEP in Jadad score at 2 or less (IV fixed-effect pooled OR = 1.31 [(95 percent CI 0.70 to 2.46); p = 0.39) or Jadad score at least 3 (IV fixed-effect pooled OR = 1.10 [(95 percent CI 0.85 to 1.43); p = 0.47). There was no significant heterogeneity in either Jadad score at 2 or less (χ^2 ^= 0.28, 1 degrees of freedom, p = 0.59) or Jadad score at least 3 (χ^2 ^= 8.11, 4 degrees of freedom, p = 0.09) (Table 3).

**Table 2:** Sensitivity-analysis of the effect of corticosteroid prophylaxis of post-ERCP pancreatitis in clinical trials

**Method**	**Patients**	**Pooled OR (95% CI)**	**p**
	**PEP**		
	
A	2632	1.13 (0.89, 1.44)	0.32
B	2328	1.20 (0.93, 1.55)	0.17
C	2247	1.10 (0.85, 1.43)	0.47
D	2448	1.13 (0.88, 1.46)	0.34
	
	**Severe PEP**		
	
A	2257	1.61 (0.74, 3.52)	0.23
B	2073	1.80 (0.68, 4.72)	0.23
C	1872	1.35 (0.47, 3.91)	0.58
D	2073	1.80 (0.68, 4.72)	0.23
	
	**Post-ERCP hyperamylasemia**		
	
A	412	0.92 (0.57, 1.48)	0.73
B	228	0.87 (0.52, 1.46)	0.60
C	228	0.87 (0.52, 1.46)	0.60
D	228	0.87 (0.52, 1.46)	0.60

A: all included trials [18–24]

B: we excluded the trials that the allocation concealment was inadequate or unclear [18, 24]

C: we excluded the trials that the blindness was unadopted [18, 21]

D: we excluded the trials which published in the form of abstract [18]

Severe PEP was reported in five trials [18,20-23]. These five trials included 2257 patients with 26 patients suffering from severe PEP (16 (1.4%) in corticosteroid treatment group and 10 (0.9%) in control group). The Q test of heterogeneity between studies was not significant (χ^2 ^= 1.34, 3 degree of freedom, p = 0.72). The meta-analysis did not indicate association between corticosteroid use and reduction of severe PEP [IV fixed-effect pooled OR 1.61 (95 percent CI 0.74 to 3.52); p = 0.23] (Figure 2, Table 2). When studies were stratified by setting, there was no significant reduction in severe PEP in either single centre studies (IV fixed-effect pooled OR 1.99 (95 percent CI 0.70 to 5.67); p = 0.20) or multicentre studies (IV fixed-effect pooled OR 1.21 (95 percent CI 0.37 to 4.00); p = 0.75) (Table 3). There was no significant heterogeneity in single centre studies (χ^2 ^= 1.12, 2 degrees of freedom, p = 0.57). Only one multicenter study reported severe PEP, so the result of heterogeneity was unavailable. When stratified by Jadad score, there was no significant reduction in severe PEP in Jadad score at 2 or less (IV fixed-effect pooled OR = 1.98 [(95 percent CI 0.62 to 6.34); p = 0.25) or Jadad score at least 3 (IV fixed-effect pooled OR = 1.35 [(95 percent CI 0.47 to 3.91); p = 0.58). There was no significant heterogeneity in either Jadad score at 2 or less (χ^2 ^= 1.12, 1 degrees of freedom, p = 0.29) or Jadad score at least 3 (χ^2 ^= 0.14, 1 degrees of freedom, p = 0.71) (Table 3).

**Table 3:** Subgroup analysis of the effect of corticosteroid prophylaxis of post-ERCP pancreatitis in clinical trials

					**Heterogeneity**		
					
**Variable**	**Patients**	**Pooled OR (95% CI)**	***Z***	**p**	**χ^2^**	**d.f.**	**p**
	**PEP**						
	
Jadad score							
≥ 3	2247	1.10 (0.85, 1.43)	0.72	0.47	8.11	4	0.09
≤ 2	385	1.31 (0.70, 2.46)	0.85	0.39	0.28	1	0.59
Setting							
Single centre	1034	1.04 (0.66, 1.64)	0.16	0.87	4.75	3	0.19
Multicentre	1598	1.17 (0.88, 1.56)	1.07	0.29	3.84	2	0.15
	
	**Severe PEP**						
	
Jadad score							
≥ 3	1872	1.35 (0.47, 3.91)	0.55	0.58	0.14	1	0.71
≤ 2	385	1.98 (0.62, 6.34)	1.14	0.25	1.12	1	0.29
Setting							
Single centre	914	1.99 (0.70, 5.67)	1.28	0.20	1.12	2	0.57
Multicentre	1343	1.21 (0.37, 4.00)	0.32	0.75	NA	NA	NA
	
	**Post-ERCP hyperamylasemia**						
	
Jadad score							
≥ 3	228	0.87 (0.52, 1.46)	0.53	0.60	NA	NA	NA
≤ 2	184	1.24 (0.37, 4.22)	0.35	0.73	NA	NA	NA
Setting							
Single centre	184	1.24 (0.37, 4.22)	0.35	0.73	NA	NA	NA
Multicentre	228	0.87 (0.52, 1.46)	0.53	0.60	NA	NA	NA

NA = not applicable

In addition, case-fatality ratio of PEP in these trials was extracted with report of case-fatality ratio in three trials [20-22]. The three trials included 958 patients but with zero death in corticosteroid group and in placebo group. So, further evaluation of case-fatality ratio of corticosteroid in prophylaxis of PEP is required in the future.

### Secondary outcome

Both post-ERCP hyperamylasemia and abdominal pain were considered as secondary outcome in the report. For post-ERCP hyperamylasemia, data were derived from two RCTs [18,22]. These trials included 412 patients with 126 patients suffering from post-ERCP hyperamylasemia. Among these patients, 61 (29.9%) patients were treated with corticosteroid and 65 (31.3%) patients with placebo. The Q test of heterogeneity of effect sizes was not significant (χ^2 ^= 0.28, 1 degree of freedom, p = 0.60). Although the post-ERCP hyperamylasemia was noted in 29.9% of patients with corticosteroid and 31.3% of control patients, the results of the meta-analysis indicated no significant association between the use of corticosteroid and reduction of post-ERCP hyperamylasemia [IV fixed-effect pooled OR 0.92 (95 percent CI 0.57 to 1.48); p = 0.73] (Figure 2, Table 2). The subgroup analysis of post-ERCP hyperamylasemia was shown in Table 3.

We were unable to identify any data on post-ERCP abdominal pain in these trials.

### Sensitivity-analysis

Three different methods were employed to perform sensitivity-analysis of these trials. First, we excluded the trials that allocation concealment was inadequate or unclear [18,24]. Second, we excluded the trials that blindness was not adopted [18,21]. Third, we excluded the trials which published in abstract [18]. As shown in Table 2, the overall estimates were virtually identical and the confidence intervals were similar between the sensitivity-analysis and the meta-analysis.

### Publication bias

**Figure 3:**
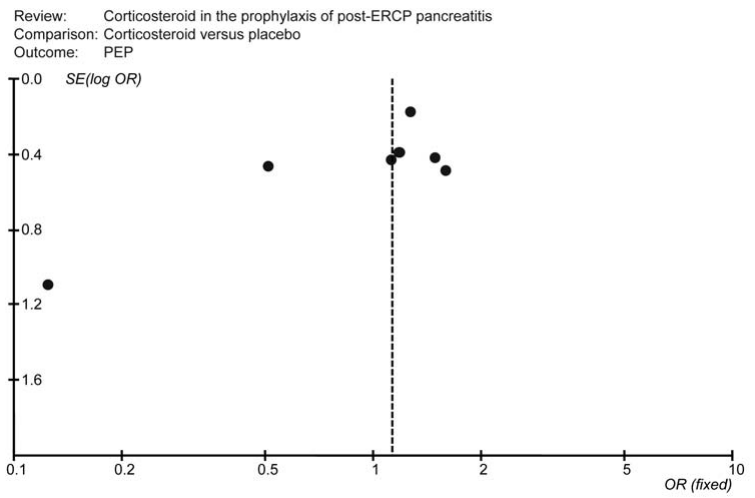
A funnel plot to explore publication bias.

Publication bias was assessed for all pooled ORs with confidence intervals using Begg's test [29,30]. This is a scatter plot with the treatment effects estimated from individual studies on horizontal axis (OR) and the standard error of the estimate on vertical axis (S.E [log OR]). In the Figure 3, all studies were laid within the 95% CI and were uniformly distributed around the vertical axis, indicating no publication bias (Begg's test showed that the p value was 0.133).

### Adverse effect

Adverse effects of corticosteroid were evaluated in current study. We found that three reports indicated different adverse effects of corticosteroid. In one report [20], there are 22 patients with adverse effects. 9 patients were in corticosteroid group and thirteen patients in placebo group. Patients in corticosteroid group had hemorrhage in four cases, cholangitis in three cases and acute cholecystis in two cases. The thirteen patients in the placebo group had the similar adverse events: hemorrhage in six, cholangitis in three, acute cholecystis in four cases. In another report from United States [19], common symptoms such as abdominal pain, vomiting, and fever or chills were reported in both groups with no significant difference between corticosteroid treatment and placebo groups. It was reported by Sherman et al. [23] that the commonest adverse effects in corticosteroid group (i.e., nausea, emesis, sweating, and rash) were also observed with similar frequency in placebo group (5.23% vs. 3.39%, corticosteroid and placebo groups respectively; p = 0.14). Therefore, it was concluded that there was no significant correlation between the use of corticosteroid and adverse effects.

## Discussion

Acute pancreatitis is the most frequent and serious complication of ERCP and affords ample opportunity to evaluate potential prophylactic therapy prior to pancreatic injury. There were several attempts intended to find therapeutic agents to prevent post-ERCP pancreatitis. These agents include calcitonin, aprotonin, glucogan, somatostatin, corticosteroid and gabexate mesilate [5-12,21-24,31-33]. Corticosteroid was proposed to use as prophylactic agent for ERCP due to its anti-inflammatory property and autodigestion theory of post-ERCP pancreatitis. Since the report of Dr. Weiner's retrospective study of therapeutic use of corticosteroid prior to ERCP procedure, several prospective randomized clinical trials have been conducted. Only few studies indicated a beneficial effect of corticosteroid on prevention of post-ERCP pancreatitis while most clinical trials did not recommend prophylactic use of corticosteroid during ERCP procedure. Moreover, some clinical trials have to be terminated after mid-term evaluation due to increase in incidence of pancreatitis in corticosteroid group compared to that of control group [22,23]. The warrant is due to the report that corticosteroid induced pancreatitis in susceptible patients when administered chronically for non-pancreatic diseases. However, proof of this causal relationship is limited [34-36]. On the other hand, it has been reported that corticosteroid successfully treat acute pancreatitis [37].

The first study of corticosteroid use in ERCP was conducted in United States in 1995 and the study involved in patients with oral or intravenous corticosteroid in patients with iodine-sensitivity to reduce incidence of PEP [17]. In this report, it was found that corticosteroid was able to reduce pancreatitis after ERCP as compared to the placebo (the occurrences of PEP were 4.6% versus 7.4% respectively). The encouraging results of the retrospective survey of Weiner et al. [17] led to the increasing interest in corticosteroid as a promisable pharmacologic agent for prophylactic prevention of PEP. However, except the study of Kwanngern et al. [24] in 2005, all others studies revealed a negative conclusion of prophylactic use of corticosteroid for ERCP [18-23]. Therefore, a meta-analysis is needed to evaluate publication bias and conclusion of the results.

Current study collected seven RCTs [18-24], which were published in the world with different languages. The effectiveness and safety of corticosteroid in the prophylaxis of post-ERCP pancreatitis were evaluated. The meta-analysis showed that occurrences of PEP [OR = 1.13, 95%CI (0.89~1.44), p = 0.32], severe PEP [OR = 1.61, 95%CI (0.74~3.52), p = 0.23], and post-ERCP hyperamylasemia [OR = 0.92, 95%CI (0.57~1.48), p = 0.73] did not correlate with the prophylactic use of corticosteroid. The results of meta-analysis indicated that corticosteroid could not prevent pancreatic injury after ERCP. Moreover, a trend toward higher rates of pancreatitis in corticosteroid treated group suggested that administration of corticosteroid might be hazardous. Furthermore, there was no association between the prophylactic use of corticosteroid and adverse effects although it was reported in three RCTs [19,20,23]. The quality of these RCTs was examined according to the Jadad score [25], and it was found that the results of meta-analysis were consistent with the sensitivity-analysis. Therefore, there was no publication bias in these RCTs.

However, what could be the difference among these RCTs and conclusions? Meta-analysis of these RCTs indicated that there were differences in experimental design (retrospective vs. prospective), forms of corticosteroid (hydrocortisone vs. methylprednisolone or prednisone), doses of corticosteroid (80 mg to 125 mg), routes of administration (oral administration vs. intravenous injection) and time of administration (immediate vs. 15, 3 or 1 hour before ERCP). Retrospective study could be different from prospective study due to patient recruitment and treatment schedule. In addition, the prospective study indicating positive recommendation of corticosteroid application in ERCP had different dose and schedule of corticosteroid (100 mg hydrocortisone intravenously one hour before ERCP) [24] than other RCTs that concluded negative result of corticosteroid use in ERCP (either 120 mg or 100 mg intravenously immediately before ERCP or 40 mg orally 15 and 3 hours before ERCP) [18-23]. Therefore, further studies with the standard administration of corticosteroids in ERCP are needed to provide solid evidence regarding its effectiveness in the prevention of post-ERCP pancreatitis.

## Conclusion

In conclusion, the present study shows no statistically significant benefit of prophylactic corticosteroid use for prevention of PEP. Therefore, the use of corticosteroids in the prophylaxis of PEP is not routinely recommended.

## Abbreviations

PEP: Post-endoscopic retrograde cholangiopancreatography pancreatitis. ERCP: Endoscopic retrograde cholangiopancreatography. OR: Odds ratio. CI: Confidence intervals. CBMdisc: China Biological Medicine Database.

## Competing interests

The author(s) declare that they have no competing interests.

## Authors' contributions

MZ: planning, data collection, study design and analysis, drafting and revising the manuscript. JB: data collection, study design and statistic analysis. BY: study design and analysis, and help to draft the manuscript. FL: study design and analysis, and help to draft the manuscript. JY: data collection, study design and statistic analysis. ML: data collection, study design and statistic analysis. YG: conceiving of the study, participated in its design and helping to draft and insightful review of the manuscript. YC: conceiving of the study, participated in its design and helping to draft the manuscript. All authors read and approved the final manuscript.

## Pre-publication history

The pre-publication history for this paper can be accessed here:


